# A Decoding Prediction Model of Flexion and Extension of Left and Right Feet from Electroencephalogram

**DOI:** 10.3390/bs12080285

**Published:** 2022-08-13

**Authors:** Abeer Abdulaziz AlArfaj, Hanan A. Hosni Mahmoud, Alaaeldin M. Hafez

**Affiliations:** 1 Department of Computer Sciences, College of Computer and Information Sciences, Princess Nourah bint Abdulrahman University, P.O. Box 84428, Riyadh 11671, Saudi Arabia; 2 Department of Information Systems, College of Computer and Information Sciences, King Saud University, Riyadh 84412, Saudi Arabia

**Keywords:** machine learning, transfer learning, motor function therapy

## Abstract

Detection of limb motor functions utilizing brain signals is a significant technique in the brain signal gain model (BSM) that can be effectively employed in various biomedical applications. Our research presents a novel technique for prediction of feet motor functions by applying a deep learning model with cascading transfer learning technique to use the electroencephalogram (EEG) in the training stage. Our research deduces the electroencephalogram data (EEG) of stroke incidence to propose functioning high-tech interfaces for predicting left and right foot motor functions. This paper presents a transfer learning with several source input domains to serve a target domain with small input size. Transfer learning can reduce the learning curve effectively. The correctness of the presented model is evaluated by the abilities of motor functions in the detection of left and right feet. Extensive experiments were performed and proved that a higher accuracy was reached by the introduced BSM-EEG neural network with transfer learning. The prediction of the model accomplished 97.5% with less CPU time. These accurate results confirm that the BSM-EEG neural model has the ability to predict motor functions for brain-injured stroke therapy.

## 1. Introduction

Most patients with stroke incidence have motor function deficiency in both left and right feet [1], causing a substantial loss of motor occupation and daily activities [1,2,3]. Stroke therapy tends to stimulate UE motor recovery and restore motor function of both feet. A main rehabilitation process is the understanding of the EEG signals to supply a non-invasive solution for the brain signal gain model (BSM), employed in all EEG signal models. BSM systems include the following steps: EEG reading, image processing, and controller [1,2,3,4]. Motor function of left and right feet target the objects in the surroundings [4,5].

The brain signal gain model (BSM) is a learning model that can capture EEG signals and convert them into motion function. BSMs are extensively found in brain-injured therapy cases. The brain signals lead to a non-intrusive answer for the BSM. BSM models the steady state of the EEG signals for motor functions feedback systems [5,6]. the images contain disparities of muscles motivated by the brain signals [7,8,9,10,11]. In our research, EEGs are captured from stroke patient cases with motor function disabilities for stroke patients. BSM systems can stimulate the motor function-lacking body part to regain the nerves of the injured parts (left and right feet in our case). Deep learning models are usually applied for BSM schemes, spatial feature selection, classification, and recognition models [10,11,12,13,14]. The researchers in [15] presented a support vector machine to classify motor signals from images. The researchers in [16] introduced the score prediction technique, and conquered accurate classification [17]. EEG signals are investigated and used in a deep learning prediction model. This model outperformed previous models especially on large datasets. Deep learning models can label properties without geometrical engineering. This defines the neural structure systems as feature selection for EEG brain signals using BSM. Current models operated deep learning systems to capture deep features. The researchers in [15] introduced a neural network with an auto-encoder with higher classification precision than prior models on the BSM-2b sets. Researchers in [16] presented a belief deep learning prediction model using the Boltzmann model. Researchers in [17] presented the envelope map of EEG signals by employing the Hilbert technique and constructed a motor imagery-based BSM prediction deep model. They employed the model to the BSM EEG-2 dataset and exhausted the most progressive prediction accuracy stated. Researchers in [16] utilized a deep learning model depiction of multiple channel EEG signal to enhance the accuracy. The researchers in [17] built 3D feature vectors of the EEG data with a parallel CNN model. The model in [18] attained high accuracy. Deep learning techniques use EEG feature mining and achieve higher precision [18,19,20,21]. However, feature mining becomes difficult due to the medical state of stroke incident cases especially for the EEG, since capturing is hard with an effect on large-size databases. The usage of these systems for motor function spatial studies in stroke cases is limited. Our model incorporates transfer learning methodologies to efficiently reduce the size of the required training set [22,23,24,25]. Features used by transfer learning utilize incident similarities and by sub-parameter inheritance [25,26,27,28]. These parameters can be reused in a reduced dataset and can increase the effectiveness of the EEGs feature learning models [28,29,30,31,32,33]. Our research contributions are summarized as depicted below:

○ Designing a deep learning neural system with a number of additional modules and cascading transfer learning stages.
○ Improving the precision of the BSM system for the prediction of motor functions for stroke patients from their EEG signals.
○ Proposing an extension to the Dense-Net using parameter tuning and transfer learning (BSM-EEG).
○ Confirming the accuracy of the proposed model by performing a comparison to similar published models.

The remainder of this paper is organized as follows. The dataset description is presented in Section 2. The proposed model with transfer learning is presented in Section 3. The experiments and performance comparison are depicted in Section 4. Section 5 concludes the work.

## 2. Materials and Methods

### 2.1. Data Description

**Figure 1:**
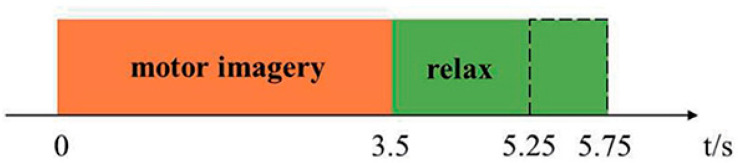
EEG signal recording versus time in seconds.

The dataset contains the EEG data of 100 cases (an average of 20 different motor function for each case). The EEG signal per case continues for 3.5 s as depicted in Figure 1. The public dataset has records of the EEG signals of the patient while he is doing different motor functions of his left and right feet. Then, we let him relax for two seconds. The public dataset can be accessed by registration from https://www.bbci.de/competition/iv/#dataset2a accessed on 12 May 2022 and https://www.bbci.de/competition/iii/#data_set_iiia accessed on 15 May 2022.

**Figure 2:**
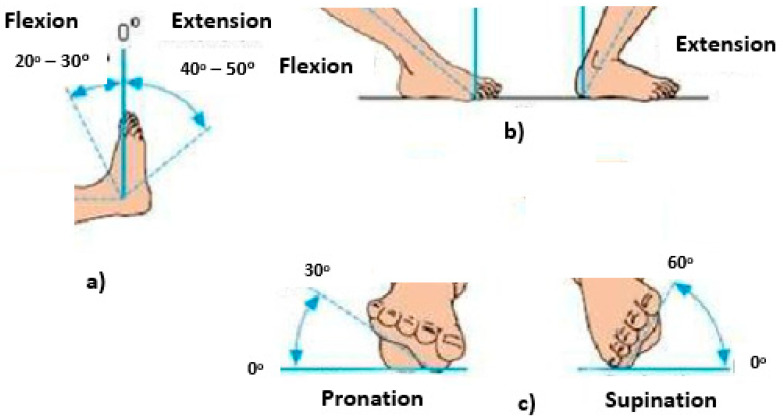
The motor functions of the foot (**a**–**c**).

These data items are recorded and labeled in a public dataset that we utilized for our experiments [15]. The motor functions of the foot are depicted in Figure 2. Figure 2a displays the flexion and extension of the foot in the ranges of 0–30 and 0–50 respectively. Figure 2b displays the flexion and extension of the foot in vertical position. Figure 2c displays the pronation and supination of the foot in the ranges of 0–30 and 0–60 respectively The statistics of the motor function of left and right feet data are shown in Table 1. These data are extracted from the public dataset in [15]. The recorded data include the foot with all the reflexes. The dataset statistics are depicted in Table 1 and Table 2.

### 2.2. Preprocessing Task

EEG data were processed through Matlab with the toolboxes BraSig 2.3.0 and EEGProc 13.1.0, Matlab Inc. (Asheboro, NC, USA).

The four preprocessing steps were as follows:

1. Removal of noisy channels, we erased the channel AFz as it is impacted by eye blinks.
2. Removal of static outliers using ICA using EEG signal with frequency 0.5–60 Hz to capture the outliers. We erased static outliers by applying the zero-phase band-pass filter using independent component analysis. We concentrated the EEG channels with principal component analysis and kept only components that capture 98% of the variations of the data.
3. Detection of attempts with transitory artefacts (EEG signal from 0.5–60 Hz). We distinguished transitory artefacts using EEGProc and signaled attempts for denial with values more than −90 μV or less than 90 μV.
4. Removal of static and transitory artefacts (computed from step 2 and 3) from the EEG signal in the range of 0.5 Hz to 5 Hz [34,35,36,37].

A total of 120 attempts were recorded for each patient as depicted from the data. We used K-fold validation dividing the data into 70%, 15%, and 15% for training, testing, and validation respectively. 

## 3. Deep Learning Phase: The Proposed BSM-EEG Model

BSM-EEG is a deep learning model with cascading transfer learning model for handling EEG signals through training on EEG signals of healthy cases and the motor functions associated with them. The prediction phase is to predict the motor function from the EEG of the brain-injured cases.

### 3.1. Methodology

**Figure 3:**
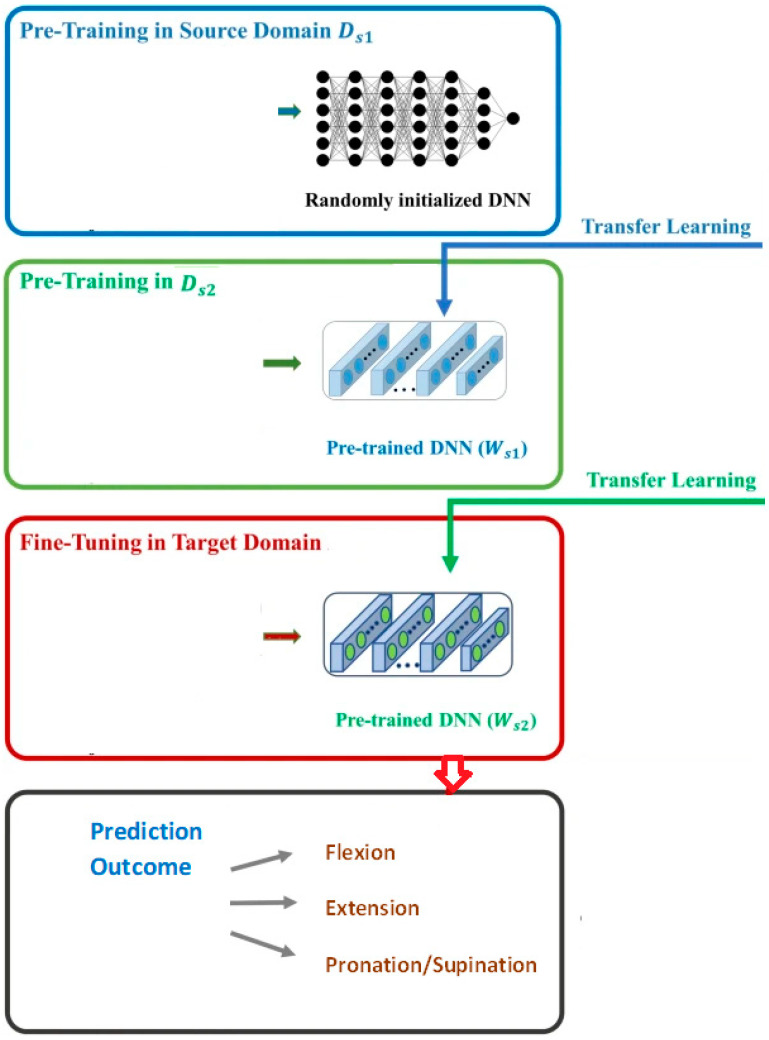
Methodology to obtain deep learning models for transfer learning (the flow diagram of the BSM-EEG model).

Our methodology aims to achieve a learning transfer model from other deep learning models that are trained on other motor functions for brain-injured cases, namely as upper limb movements (source domain 1) [7] and knee movements (source domain 2) [5]. Each source domain contains an average of 30,000 different labeled motor function EEG. To do so, we employed several input domains $D_{s}$ to get the suitable learning transfer models. Figure 3 displays the phases to accomplish this objective. We can have several input domains. For each domain $D_{si}$, an optimal deep neural network was attained via Bayesian procedure. The optimization module output the parts of $D_{si}$ which was utilized to train the final deep learning model. The training data of the transfer learning model were chosen due to its prediction accuracy over the labeled target domain. The flow diagram of the proposed model is depicted in Figure 3.

The presented model comprises four stages:

(1) Transfer training in input domain $D_{s1}$ utilizing upper limb labeled, motor function labeled EEG signals. A deep neural network was trained to learn the EEG signals for upper limb motor functions. The structure of this deep learning network was optimized to realize higher accuracy.
(2) Unsupervised training phase on the same dataset $D_{s1}$ utilizing non-labeled data items from $D_{s1}$ and from other data items not included in $D_{s1}$. We adjusted the pre-trained deep learning model from first phase by utilizing the same neural weights.
(3) Fine-tuning in the target input domain $D_{T}$ using 271 labeled EEGs with their desired lower limb motor functions.

### 3.2. Architecture 

To choose the suitable deep learning model with the correct weights is the Bayesian selection process [21]. Random Bayesian selection of the convolutional weight space leads to higher accuracy. In a Bayesian optimization model, the parameters of the deep learning model are computed as the optimization of an objective function. The objective function’s goal is to optimize the loss function of the deep learning model by adjusting the selection space.

In this paper, we present several Bayesian procedures to obtain the deep learning model that achieves better performance on the source input domains. The training process phases is depicted as follows.

The first phase was to train an initial model $DL_{i}$ with arbitrary preliminary parameters and optimize a loss function $L_{i}$ for the source input domains $D_{si}$ in each source domain. 

For each source $D_{s}$, an optimized deep learning (DL) neural network was achieved via Bayesian optimizer. During this process, the source input domain was divided into a training subset and validation subset. The DL model was verified based on the transfer learning performance using the target input domains. This Bayesian optimizer was applied on all source domain data. The model with the highest performance was chosen by computing the performance metrics on transfer learning functions using a function. 

The last step was to validate the usefulness of prior models for transfer learning optimization by utilizing all target datasets by optimizing the loss function ($L_{Bays}$) of the Bayesian optimizer.

(1) $$\mathrm{Min}\;L_{Bays}\;\left[DL_{i}\;\underset{\;}{\Rightarrow }\;DL_{j\;}\right]\;\forall \;i\;\neq j\;$$

where $\underset{\;}{\Rightarrow }$ is a transfer learning operator. $DL_{i}$ is a deep learning model and is trained with only a single source input domain and its weights are transferred to the other source domains by fine-tuning the fully connected layers in $DL_{j\;}$. The loss function Loss is calculated as a weighted (*w*) accuracy (*acc*) average and the average loss in both the learning and validation process and is computed as follows:

(2) $$Loss=w\left[\left(1-acc_{learning}\right)+Loss_{learning}]+\left(1-w\right)\;[\left(1-acc_{validation}\right)+Loss_{validation}\right]$$

**Figure 4:**
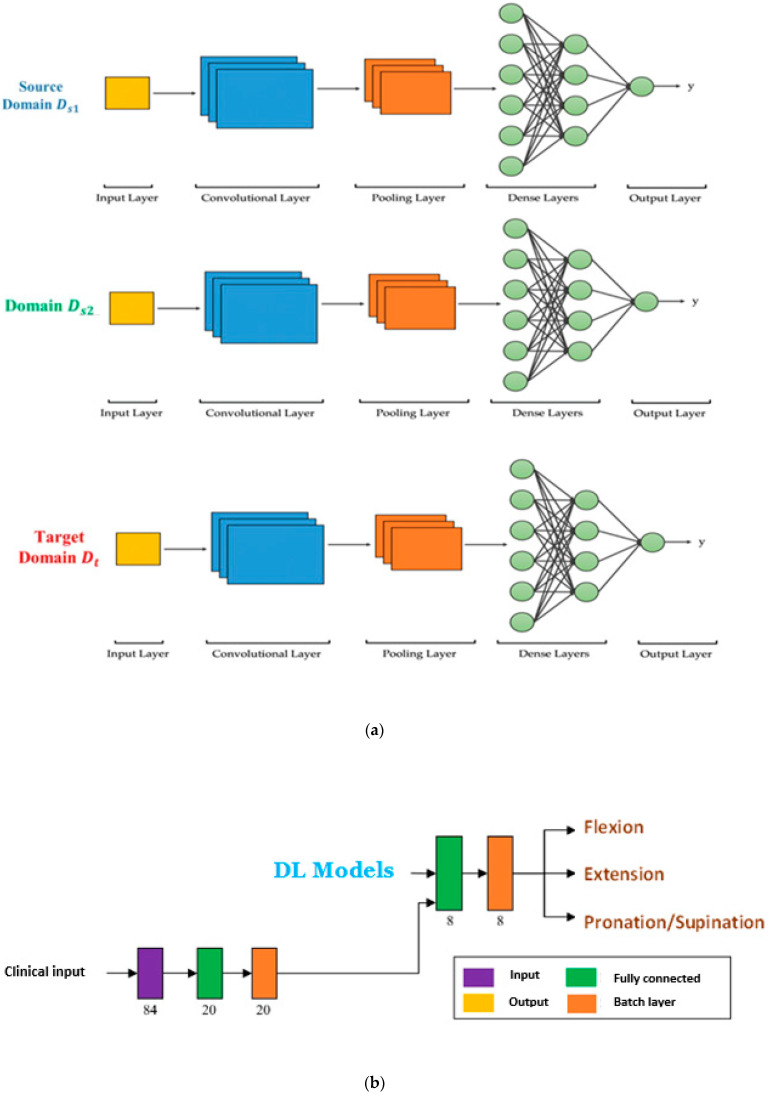
(**a**) The architecture of the transfer learning training; (**b**) the architecture of prediction from actual labeled clinical data.

The final step of this stage was a set of DL models equal to the count of source domains. The architecture of transfer learning training and prediction from actual labeled clinical data is depicted in Figure 4.

## 4. Results and the Prediction Performance

### 4.1. Training

The proposed model training was done on a Sun station CPU X6-3320 V2@ 3.60 GHz* 16 with 64 bits Linux operating system as depicted in Table 3. The deep learning model was implemented in Python 3.6.0. The method of the training was to modify the filter weights to ensure that the classified result is near to the labeled class. The utilized dataset was partitioned into three partitions. The first partition was the training subset and it included 70% of the dataset. The second partition was the validation subset and it included 15% of the dataset. The third partition was the validation subset and it included 15% of the dataset for testing the efficiency of the model. Adam optimizer was employed for fine-tuning the neural weights to minimize the loss. Table 4 depicts the hyperparameters utilized for training.

### 4.2. Experiment Setting 

**Figure 5:**
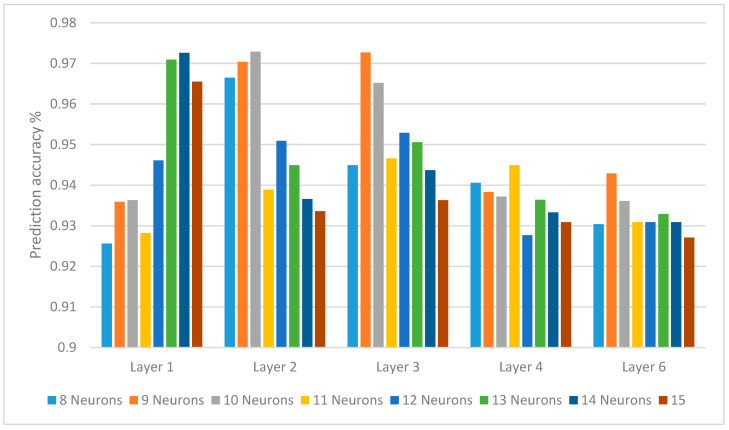
Prediction accuracy of various counts of neurons in convolutional layers.

The experimental setting included determining the number of hidden layers of the DL model and the number of neurons in each layer, number of epochs, and learning rate. To define the construction of the neural structure, hidden layers and the neurons in the different layers had to be defined. The results of various hidden layer numbers and neuron counts are depicted in Table 5 and displayed in Figure 5. The count of iterations was 1900.

**Figure 6:**
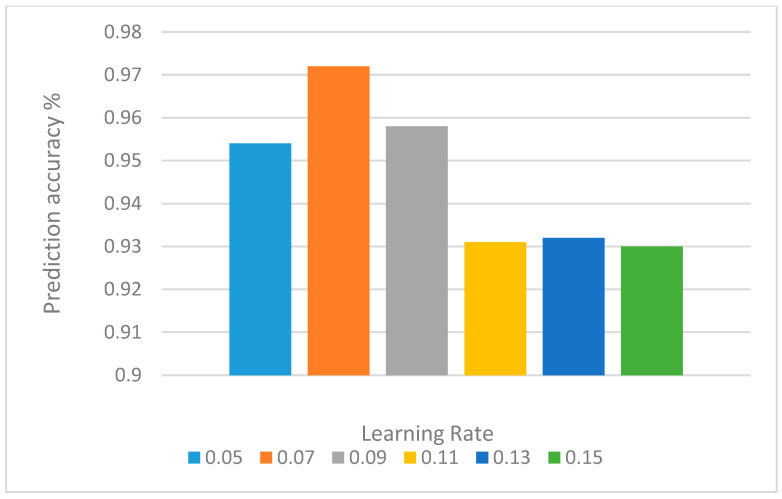
The impact of learning rate on performance.

The various learning rate also impacts the accuracy of the neural network. We tested learning rate between 0.05 and 0.15, with step of 0.02. The results of several learning rates are depicted in Table 6 and displayed in Figure 6. The results prove that the proposed model had the highest performance with learning rate equals to 0.07.

## 5. The Proposed Models with Transfer Learning from Different Domain Sources

### 5.1. Performance Metrics

To analyze the performance of the proposed model, several performance metrics were utilized, which proved the efficiency of the model in predicting foot movement from the EEG. The evaluation metrics were *recall*, f1-score, *precision*, and *accuracy* (they are defined in the following equations).

(3) $$Precision=\frac{TP}{TP+FP}$$

(4) $$Recall=\frac{TP}{TP+FN}$$

(5) $$Accuracy=\frac{TP+TN}{TP+TN+FP+FN\;}$$

(6) $$F2-Score=\frac{2\times Recall\times Precision}{Recall+precision}$$

where TP is the number of true positive predictions, TN is the number of true negative predictions, FP is the number of false positive predictions, and V is th FN number of false negative predictions.

The classification accuracy, recall, and F1-score of our model are depicted in Table 7. The mentioned table compares between the performance metrics of our model and transfer learning with one and two source domain.

### 5.2. Confusion Matrix

The confusion matrices of predicting foot movement from the EEG is depicted in Table 8, Table 9 and Table 10, which display the true label (ground truth) at the y-axis and the predicted foot movement at the x-axis. The confusion matrices are for the proposed model without transfer learning (Table 8), the proposed model with transfer learning from one source domain (Table 9), and the proposed model with transfer learning from two source domains (Table 10).

### 5.3. Time Complexity Versus Accuracy

In this research, it was essential to compute the time complexity for the deep learning model and how transfer learning could affect the training time complexity. Moreover, it was important to see the tradeoff between the deep learning model alone and the trade off when we incorporated the transfer learning for one or more sourced domains. The results are presented in Table 11 and Table 12.

### 5.4. Performance Comparison of Different Models

The experiments have a big role in determining the hidden layers and the optimized count of neuron with learning rate in accordance. The selected parameters were applied to our proposed deep learning model. We comparatively evaluated our models with other DL models with transfer learning with the same parameter settings. The compared models were BP neural [13], TransferN [19], DLN [21], CNN [27], and STL [31]. The parameter settings were the same for all the compared models. Since transfer learning models need relatively lengthy training times, the training time and prediction time of different models are shown in Table 13.

## 6. Conclusions

The goal of this research was to decode the left and right foot motor functions from EEG signals. The proposed deep learning model realized high prediction precision which can lead to a better a brain signal gain model (BSM) which can be employed in several limb assistive devices. The proposed research attained high accuracy by applying transfer learning from other source domains such as from elbow and knees source input domains. Our method realized higher accuracy of 97.4% by training through EEG signals of healthy cases performing motor feet functions. The presented classifier can be deployed in several classes of BSM as control signals for operative foot neuro pros. The research also concluded that the proposed BSM-EEG model with cascading transfer learning with deep learning can be competently employed on a small size input. 

This research indicates that the presented model can transfer learning for the same pattern. The experimental results depict that transfer learning should be incorporated in the paradigm of EEG processing. The BSM-EEG outperformed other state-of-the-art neural deep learning models in motor imagery detection. The experiments showed that we can utilize a small-sized dataset for training by incorporating feature extraction through other source domains. The mechanism of this study can be generalized by using n source domains instead of only two source domains.

## Figures and Tables

**Table 1:** The statistics of the motor function of left and right feet data.

Motor Function	Mean	Standard Deviation	Minimum	Maximum
Right foot flexion	18.9°	3.4°	0	30°
Left foot flexion	20.5°	2.68°	0	30°
Right foot extension	40.7°	5.67°	0	50°
Left foot extension	42.7°	6.3°	0	50°
Right foot pronation	25.96	2.87	0	30°
Left foot pronation	26.71	3.63	0	30°
Right foot supination	51.71	5.73	0	60°
Left foot supination	48.96	4.87	0	60°

**Table 2:** Dataset statistics (total samples of EEG signals: 2000 from 271 cases).

Foot Movement Associated with the EEG	Count
Right foot flexion	222
Left foot flexion	200
Right foot extension	208
Left foot extension	300
Right foot pronation	250
Left foot pronation	200
Right foot supination	300
Left foot supination	320

**Table 3:** Environment.

Hardware	
Processor	RAM
Sun station CPU X6-3320 V2@ 3.60 GHz^*^ 16	64 GB
**Software**	
Operating system	Simulation environment
Linux	Python 3.4 and Mat lab

**Table 4:** Hyperparameters utilized for training.

Stage	Layer	Hyperparameter Value
First Convolution	Filters	128
	Kernel size	5
	Strides	3
	Average pooling	8
Second Convolution	Filters	256
	Kernel size	4
	Average pooling	4
Third Convolution	Filters	512
	Kernel size	2
	Max pooling	2
Training Parameters	Learning rate	0.2
	Epochs	80
	Batch size	26
	Optimizer	Adam

**Table 5:** Prediction accuracy of various counts of neurons in convolutional layers.

Neuron Counts	8	9	10	11	12	13	14	15
Layer 1	0.9256	0.9359	0.9363	0.9282	0.9461	0.9709	0.9726	0.9655
Layer 2	0.9665	0.9704	0.9729	0.9389	0.9509	0.9449	0.9366	0.9336
Layer 3	0.9449	0.9727	0.9652	0.9466	0.9529	0.9506	0.9437	0.9363
Layer 4	0.9406	0.9383	0.9372	0.9449	0.9277	0.9364	0.9333	0.9309
Layer 6	0.9304	0.9429	0.9361	0.9309	0.9309	0.9329	0.9309	0.9271

**Table 6:** The impact of learning rate on performance.

Learning Rate	0.05	0.07	0.09	0.11	0.13	0.15
Accuracy	0.954	0.972	0.958	0.931	0.932	0.930

**Table 7:** Classification report of our model with transfer learning with one and two source domain model.

	Our Model with Transfer Learning with One Source Domain			Our Model with Transfer Learning with Two Source Domain		
Predicted movement	Precision	Recall	F2-score	Precision	Recall	F2-score
Right foot flexion	0.9	0.95	0.9	0.97	0.99	0.96
Left foot flexion	0.8	0.85	0.8	0.96	0.96	0.96
Right foot extension	0.94	0.85	0.91	0.92	0.96	0.97
Left foot extension	0.94	0.85	0.9	0.97	0.92	0.96
Right foot pronation	0.89	0.93	0.91	0.96	0.94	0.97
Left foot pronation	0.9	0.9	0.91	0.96	0.9	0.96
Right foot supination	0.84	0.9	0.8	0.94	0.9	0.96
Left foot supination	0.94	0.9	0.9	0.97	0.9	0.99

**Table 8:** Confusion matrix for the proposed DL model without transfer learning.

Motor Function	Right Foot Flexion	Left Foot Flexion	Right Foot Extension	Left Foot Extension	Right Foot Pronation	Left Foot Pronation	Right Foot Supination	Left Foot Supination	Total Cases
Right foot flexion	94	2	50	3	52	1	20	0	222
Left foot flexion	3	100	4	33	2	22	1	35	200
Right foot extension	20	5	107	5	30	2	18	21	208
Left foot extension	10	40	0	150	10	40	11	39	300
Right foot pronation	22	8	30	10	130	10	30	10	250
Left foot pronation	6	19	11	31	4	110	9	30	200
Right foot supination	21	0	29	10	60	5	170	5	300
Left foot supination	4	51	0	49	11	30	5	170	320

**Table 9:** Confusion matrix for the proposed DL model with transfer learning with one source domain.

Motor Function	Right Foot Flexion	Left Foot Flexion	Right Foot Extension	Left Foot Extension	Right Foot Pronation	Left Foot Pronation	Right Foot Supination	Left Foot Supination	Total Cases
Right foot flexion	184	2	10	3	12	1	10	0	222
Left foot flexion	1	170	2	10	2	9	1	5	200
Right foot extension	8	2	180	7	1	2	8	0	208
Left foot extension	2	8	0	270	1	9	3	7	300
Right foot pronation	10	1	7	10	220	1	9	2	250
Left foot pronation	1	7	2	5	1	175	2	7	200
Right foot supination	8	0	9	2	11	3	265	2	300
Left foot supination	3	10	1	11	3	9	4	280	320

**Table 10:** Confusion matrix for the proposed DL model with transfer learning with two source domains.

Motor Function	Right Foot Flexion	Left Foot Flexion	Right Foot Extension	Left Foot Extension	Right Foot Pronation	Left Foot Pronation	Right Foot Supination	Left Foot Supination	Total Cases
Right foot flexion	211	0	4	0	5	0	2	0	222
Left foot flexion	0	195	0	1	1	2	0	1	200
Right foot extension	2	0	200	0	3	1	2	0	208
Left foot extension	0	1	0	295	0	2	0	2	300
Right foot pronation	1	0	2	0	244	1	2	0	250
Left foot pronation	0	1	0	2	0	196	0	1	200
Right foot supination	2	0	1	1	2	0	292	2	300
Left foot supination	0	1	1	2	0	1	0	315	320

**Table 11:** Time complexity of the proposed model with and without transfer learning.

	Our Model with Transfer Learning with One Source Domain	Our Model with Transfer Learning with Two Source Domain
Training CPU time (h)	12:32	18:57
Classification time (s)	119.9 s	90.3 s

**Table 12:** Performance comparison of the proposed model with and without transfer learning.

Model	Average Accuracy for All Motor Functions (%)	Average Training Time (h)	Average Classification Time (s)
Our model without transfer learning	57.10%	8.1	113.1
Our model with transfer learning with one source domain	90.90%	12.9	119.9
Our model with transfer learning with two source domain	97.30%	17.3	90.3

**Table 13:** Performance comparison.

Model	BP Neural	TransferN	DLN	STL	Our Model without Transfer Learning	Our Model with Transfer Learning with One Source Domain	Our Model with Transfer Learning with Two Source Domain
Acc	0.6136	0.6443	0.6666	0.6611	0.5668	0.91	0.97
Time(s)	64	106	113	132	120	119	90.3

## Data Availability

The public dataset can be accessed by registration from https://www.bbci.de/competition/iv/#dataset2a (accessed on 1 August 2022) and https://www.bbci.de/competition/iii/#data_set_iiia (accessed on 1 August 2022).
